# Symmetric cross-entropy multi-threshold color image segmentation based on improved pelican optimization algorithm

**DOI:** 10.1371/journal.pone.0287573

**Published:** 2023-06-29

**Authors:** Chuang Zhang, Yue-Han Pei, Xiao-Xue Wang, Hong-Yu Hou, Li-Hua Fu

**Affiliations:** 1 School of Mechanical Engineering and Automation, University of Science and Technology Liaoning, Anshan, China; 2 School of Materials and Metallurgy, University of Science and Technology Liaoning, Anshan, China; 3 Chao Yang Iron & Steel Construction., Ltd. of An steel Group Corporation, Anshan, China; Universidad de Guadalajara, MEXICO

## Abstract

To address the problems of low accuracy and slow convergence of traditional multilevel image segmentation methods, a symmetric cross-entropy multilevel thresholding image segmentation method (MSIPOA) with multi-strategy improved pelican optimization algorithm is proposed for global optimization and image segmentation tasks. First, Sine chaotic mapping is used to improve the quality and distribution uniformity of the initial population. A spiral search mechanism incorporating a sine cosine optimization algorithm improves the algorithm’s search diversity, local pioneering ability, and convergence accuracy. A levy flight strategy further improves the algorithm’s ability to jump out of local minima. In this paper, 12 benchmark test functions and 8 other newer swarm intelligence algorithms are compared in terms of convergence speed and convergence accuracy to evaluate the performance of the MSIPOA algorithm. By non-parametric statistical analysis, MSIPOA shows a greater superiority over other optimization algorithms. The MSIPOA algorithm is then experimented with symmetric cross-entropy multilevel threshold image segmentation, and eight images from BSDS300 are selected as the test set to evaluate MSIPOA. According to different performance metrics and Fridman test, MSIPOA algorithm outperforms similar algorithms in global optimization and image segmentation, and the symmetric cross entropy of MSIPOA algorithm for multilevel thresholding image segmentation method can be effectively applied to multilevel thresholding image segmentation tasks.

## 1. Introduction

Image segmentation is a key step in image recognition, image analysis and a classical challenge in image processing [1, 2], and is widely used in target detection, face recognition, industry and aviation [3], among others. Its principle is the technique and process of merging pixel points with similar attributes in an image into several regions and proposing regions of interest. Currently, image segmentation methods can be roughly classified into four types: point, line, and boundary-based approaches [4], threshold-based approaches [5], region-based approaches [6], and morphology-based approaches and image segmentation algorithms formed based on specific theories [7] that have emerged in recent years. Among them, the thresholding method is becoming increasingly widely used for image segmentation because of its advantages of easy operation, high efficiency, fast processing speed, and stable performance. This method has become one of the most widely used methods in image segmentation.

As the most common method used in image segmentation, thresholding employs a parallel region segmentation technique. The segmentation method that divides an image into two major classes, background, and target, is called single-threshold segmentation, which only requires the selection of a The method of segmenting the image into multiple target and background classes is called multi-threshold segmentation, which requires the selection of multiple thresholds for processing. The segmented region is labeled [8]. However, multi-threshold segmentation of images increases exponentially in computational complexity as the number of thresholds increases, leading to problems such as low accuracy and slow convergence of traditional multi-level threshold image segmentation methods.

To solve this problem, more and more researchers are introducing swarm intelligence optimization algorithms [9] in solving image segmentation problems to improve segmentation accuracy and speed. Common threshold selection methods for multi-threshold segmentation incorporating swarm intelligence optimization algorithms include the Otsu method [10, 11], Kapur entropy method [12, 13], fuzzy entropy [14, 15], and minimum cross entropy [16, 17]. Ma [18] et al. proposed an improved multi-threshold image segmentation method based on the whale optimization algorithm (RAV-WOA) using the inter-class variance (Otsu method) as the objective function. A backward learning strategy is introduced in the initialization of the RAV-WOA population, and an adaptive weighting strategy is introduced to balance the algorithm’s global search ability and local exploitation ability. The experimental results show that the segmentation results of RAV-WOA in multi-threshold image segmentation have better quality and stability than other algorithms. Qi [19] et al. proposed a new multilevel image segmentation method (MIS-XMACO) based on the population intelligence algorithm (SIA) to enhance image segmentation of COVID-19 X-rays. An improved ant colony optimization algorithm combining directed crossover (DX) and directed mutation (DM) strategies shows more stable and superior segmentation results than other models at different threshold levels. Jiang [20] et al. proposed a multilevel thresholding image segmentation method based on the Improved Sticky Mushroom Algorithm (ISMA) and symmetric cross entropy for global optimization and image segmentation tasks and achieved better results in multilevel thresholding image segmentation by elite backward learning strategy adaptive probability thresholding and other strategies. Chen [21] et al. developed an algorithm called the Poplar Optimization Algorithm (POA) to solve the continuous optimization problem, which mimics the sexual and asexual reproduction mechanisms of poplar trees, where the algorithm details the basic idea of how to perform sexual and asexual reproduction for individuals, and the experimental results show that the algorithm can effectively find the optimal threshold for image segmentation. Hussien [22] et al. proposed the VCSWOA algorithm by fusing Gaussian wandering, CMA-ES, and evolution emerging from viral swarm search (VCS), significantly improving image segmentation results compared to other swarm intelligence algorithms. Hosny [23] et al. proposed an improved coronavirus optimization algorithm to solve the image segmentation problem and applied it to the segmentation of satellite images. Experiments showed the superiority of the proposed algorithm in the image segmentation problem. Houssein [24] et al. proposed an improved golden jackal optimization algorithm (IGJO) for skin cancer classification and early diagnosis. Experimental results showed that the algorithm outperformed other alternative algorithms regarding PSNR, SSIM, FSIM, and MSE segmentation metrics, effectively solving the segmentation problem. Yu et al. [25] updated the positions of the top three wolves in the wolf pack optimization algorithm according to the differences in alphas, thus proposing the EGWO algorithm. The experimental results showed that the EGWO algorithm is reliable and effective in practical image segmentation applications.

The above improved algorithm and most of the present researchers are experimenting with image segmentation of grayscale images, while color images often have more useful information, but there is less research on this aspect of color image segmentation.

In this paper, an improved pelican optimization algorithm (MSIPOA), is proposed and applied to multi-threshold color image segmentation. First, Sine chaotic mapping is used to make the initial population distribution more uniform, and spiral predation strategy, positive cosine optimization algorithm, and levy strategy are introduced to improve the ability of MSIPOA to jump out of local optimum. The convergence speed and accuracy of MSIPOA were verified by a total of 12 test functions with single and multiple peaks. Combining MSIPOA with symmetric cross-entropy multi-threshold segmentation effectively improves the accuracy and speed of multi-threshold image segmentation. Eight color images from the Berkeley University BSDS300 image segmentation test set were selected for the study. The experimental results show that the MSIPOA symmetric cross-entropy-based multilevel thresholding image segmentation method outperforms other swarm intelligence optimization algorithms in FSIM, SSIM, and PSNR. Therefore, the contribution of this paper is as follows:

(1) The MSIPOA algorithm is proposed for the characteristics that the POA algorithm converges slowly and quickly falls into the local optimum. Twelve test functions fully demonstrate the convergence ability of the MSIPOA algorithm.

(2) A multi-level thresholding image segmentation method based on MSIPOA symmetric cross-entropy is used for multi-threshold color image segmentation. The experimental results show that the method outperforms other swarm intelligence optimization algorithms in FSIM, SSIM, and PSNR test metrics.

The rest of this paper is organized as follows. In Section 2, the basic pelican optimization algorithm principles are presented. Section 3 details the improvement strategy of MSIOIA and compares the effect with six new algorithms in 12 test functions to verify the superiority of the MSIPOA algorithm. Section 4 compares the effectiveness of MSIPOA-based symmetric cross-entropy multilevel thresholding image segmentation methods on FSIM, SSIM, and PSNR with other swarm intelligence optimization algorithms to verify the effectiveness of MSIPOA for multi-threshold image segmentation. The conclusions of this paper are presented in Section 5.

## 2. Pelican Optimization Algorithm

The Pelican Optimization Algorithm (POA) [26] was proposed by Pavel Trojovský and Mohammad Dehghani in 2022, which simulates the natural behavior of pelicans during the hunting process, which is divided into two main phases: the approaching prey phase and the surface flight phase. The mathematical models developed by POA depending on the hunting stage are as follows:

### 2.1. Moving towards prey (exploration phase)

In the first stage of Pelican’s optimization algorithm, it randomly determines the location of the prey and then moves towards this determined area. The mathematical expression for the behavior of the pelican during this phase is as follows:

$$P_{i}=X_{k},i=1,2,\ldots ,N,k=1,2,\ldots ,N$$ (1)

$$x_{i,j}^{P1}=\{\begin{array}{cc} x_{i,j}+rand\cdot (p_{j}-I\cdot x_{i,j}), & F_{p}<F_{i}\\ x_{i,j}+rand\cdot (x_{i,j}-p_{j}), & else, \end{array}$$ (2)

$$X_{i}=\{\begin{array}{cc} X_{i}^{P_{1}}, & F_{i}^{P_{1}}<F_{i};\\ X_{i}, & else, \end{array}$$ (3)

Where *P*_i_ is the location of the prey selected by the *i* pelican; *F*_*i*_ is the value of the objective function, i.e., the value of the degree of adaptation; *k* is a random natural number belonging to [1,*N*]; $x_{i,j}^{P1}$ is the new state of the *i* pelican in the *j* dimension; $F_{i}^{p_{1}}$ is the adaptation value corresponding to it. *rand* represents a random number of [0,1] and, in addition, the value of *I* is either 1 or 2. *rand* and *I* are random numbers used to generate random POA behavior in search and update.

### 2.2. Winging on the water surface (exploitation phase)

In the second stage, when the pelicans reach the surface, they spread their wings on the water and move the fish upwards before placing the prey in their throat pockets. This strategy of surface flight by pelicans allows them to catch more fish in the area being attacked. Modeling this behavioral process of the pelican allows the POA algorithm to converge to a better location in the hunting area, which increases the local search capability and exploitation of the POA algorithm. From a mathematical point of view, the algorithm must check the positions near the pelican position so that the algorithm can converge to a better position. The mathematical expression for the second stage is as follows:

$$x_{i,j}^{P_{2}}=x_{i,j}+R\cdot (1-\frac{t}{T})\cdot (2\cdot rand-1)\cdot x_{i,j}$$ (4)

$$X_{i}=\{\begin{array}{cc} X_{i}^{P_{2}}, & F_{i}^{P_{2}}<F_{i};\\ X_{i}, & else, \end{array}$$ (5)

where *t* is the current number of iterations; *T* is the maximum number of iterations; *R* is a constant taking the value of 0.2; $x_{i,j}^{P_{2}}$ is the new state of the *i* pelican in the *j* dimension in the second hunting phase; $F_{i}^{P_{2}}$ is the corresponding fitness value in the new state.

## 3. Multi-strategy improvement of pelican optimization algorithm

### 3.1. Sine chaos initialization

Instead of random initialization, chaotic mapping makes the population more uniformly distributed in the search space. The mathematical expression of the Sine chaotic mapping is as follows:

$$x_{k+1}=\frac{a}{4}\sin(\pi x_{k}),a\in (0,4]$$ (6)

### 3.2. Fusion of improved sine cosine optimization algorithms

The sine and cosine optimization algorithm [27] uses the periodic volatility of the sine and cosine functions to construct iterative equations that implement the functions of two threads of global search and local exploitation. The perturbation is applied, and the solution set is updated by this brief update iterative equation. The specific iterative equations are classified into the following two types sine iterative or iterative cosine equations.

$$X_{i}^{j}(t+1)=\{\begin{array}{cc} X_{i}^{j}(t)+r_{1}\cdot \sin(r_{2})\cdot |r_{3}P_{best}(t)-X_{i}^{j}(t)|, & r_{4}>0.5,\\ X_{i}^{j}(t)+r_{1}\cdot \cos(r_{2})\cdot |r_{3}P_{best}(t)-X_{i}^{j}(t)|, & r_{4}<0.5, \end{array}$$ (7)

where *t* is the number of current iterations and $X_{i}^{j}(t)$ denotes the component of the position of individual *i* in dimension *j* at the *t* iteration; *r*_1_,*r*_4_ is a random number of [0,1]; *r*_2_ is a random number of [0,2*π*]; *r*_3_∈(0,+∞); *P*_*best*_(*t*) is the optimal solution position at the *t* iteration.

Inspired by the spiral predation mechanism of the whale optimization algorithm [28], the method was introduced into the pelican optimization algorithm so that it gradually approaches the prey in a spiral manner during the approaching prey phase to expand the search range and increase the global search capability. And combining this method with the sine cosine optimization algorithm, the first stage mathematical expression of the pelican optimization algorithm after fusing these two strategies is as follows:

$$x_{i,j}^{P1}=\{\begin{array}{cc} e^{z\cdot l}\cdot \cos(2\pi l)\cdot x_{i,j}+r_{1}\cdot \sin(r_{2})\cdot |r_{3}\cdot x_{i,j}-p_{j}|, & F_{p}<F_{i};\\ e^{z\cdot l}\cdot \cos(2\pi l)\cdot x_{i,j}+r_{1}\cdot \cos(r_{2})\cdot |r_{3}\cdot x_{i,j}-p_{j}|, & else, \end{array}$$ (8)

### 3.3. Introduction of levy flight mechanism

Levy flight strategy is a very effective mathematical method for providing levy distributed random factors. A levy flight strategy is introduced to enhance the ability of the Pelican optimization algorithm to jump out of the local optimum. Levy flight expression is as follows:

$$\sigma ={\left[\frac{\Gamma (1+\beta )\sin(\frac{\pi \beta }{2})}{\Gamma (\frac{1+\beta }{2})\beta \cdot 2^{\frac{\beta -1}{2}}}\right]}^{\frac{1}{\beta }}$$ (9)

$$u∼N(0,\sigma^{2}),v∼N(0,1)$$ (10)

$$levy(x)=0.01\times \frac{u\cdot rand}{{\left|v\right|}^{\frac{1}{\beta }}}$$ (11)

The mathematical expression of the second stage of the pelican optimization algorithm after incorporating levy flights is as follows:

$$x_{i,j}^{P_{2}}=levy\cdot x_{i,j}+R\cdot (1-\frac{t}{T})\cdot (2\cdot rand-1)\cdot x_{i,j}$$ (12)

**Fig 1:**
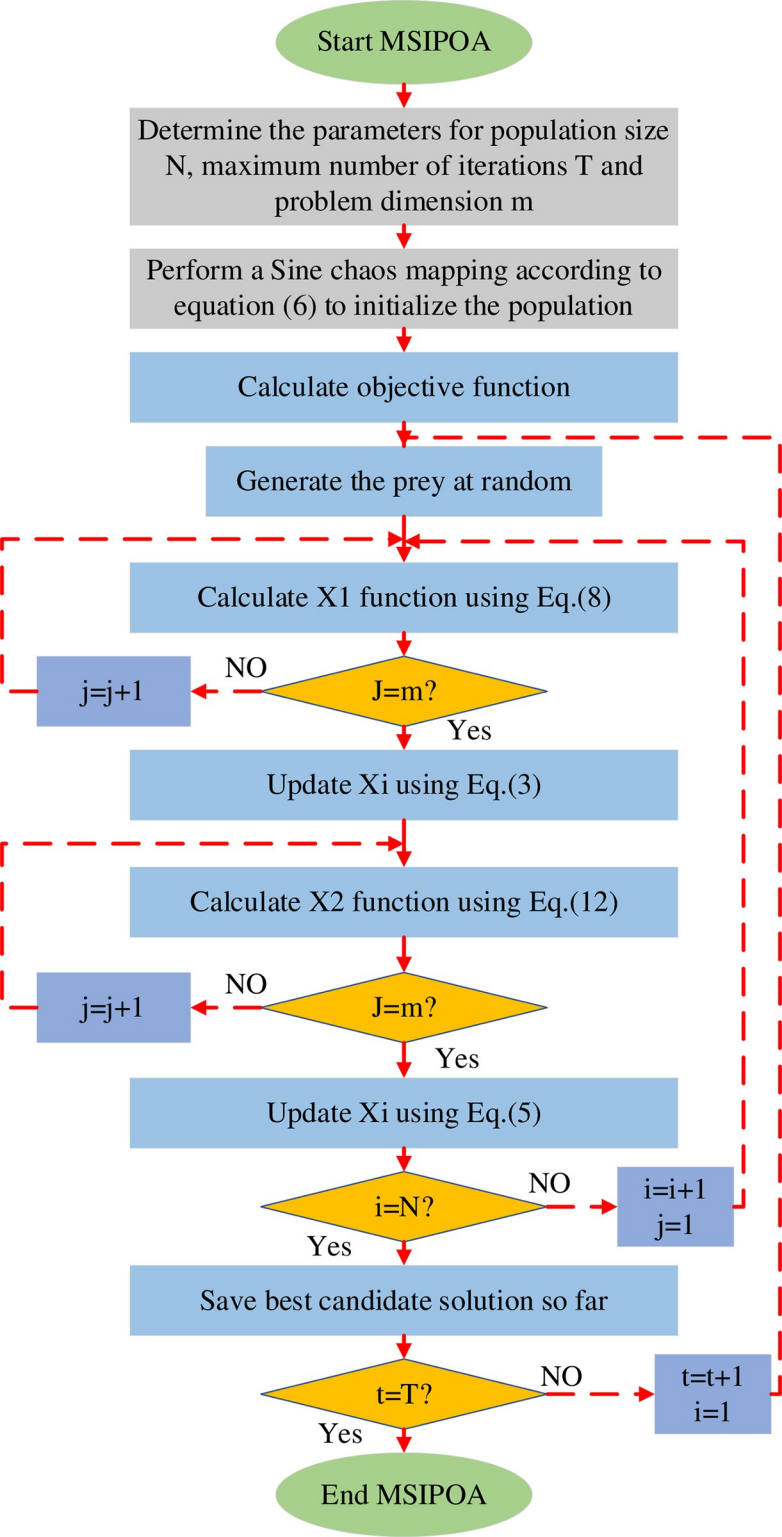
MSIPOA algorithm flow chart.

The flow chart of the MSIPOA algorithm when solving the problem is shown in Fig 1.

### 3.4. MSIPOA performance test

**Fig 2:**
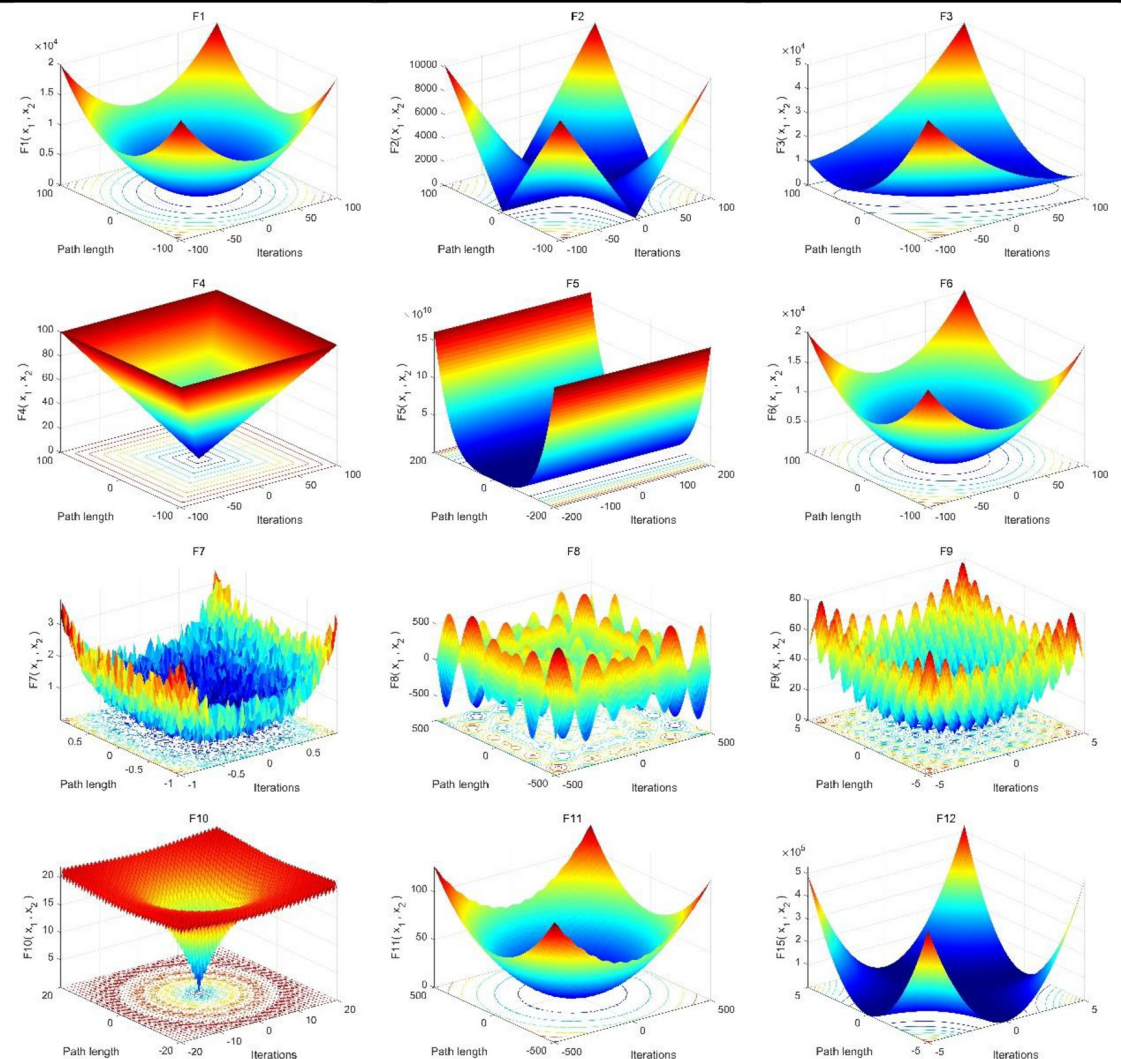
Visualization of 12 benchmarking functions.

The MSIPOA algorithm was compared with the Pelican Optimization Algorithm (POA), the Whale Optimization Algorithm (WOA), the Sand Cat Swarm Optimization Algorithm (SCSO) [29], the Dung Beetle Optimization Algorithm (DBO) [30], the Hunter Prey Optimization Algorithm (HPO) [31], the Gray Wolf Optimization Algorithm (GWO) [32], Arithmetic Optimization Algorithm (AOA) [33] and Aquila Optimizer(AO) [34]. The initial population size of each algorithm is set to 30, the maximum number of iterations is 500, and the number of independent runs is 30. Since POA, SCSO, DBO, HPO, and other algorithms are relatively new swarm intelligence optimization algorithms proposed in recent years. These algorithms have been compared with some classical swarm intelligence optimization algorithms, such as Particle Swarm Optimization (PSO) algorithm, Genetic Algorithm (GA), etc., and the experimental results show that they have better performance in finding the best, so this paper will not compare with the classical intelligence algorithms. In this paper, 12 basic test functions are selected to test the performance of each algorithm. The detailed benchmark test function information is shown in Table 1, where F1~F7 are single-peak test functions, F8~F12 are multi-peak test functions, Range represents the search range of the solution, Dim is the dimensional information of the test function, *f*_min_ is the theoretical optimal value of the test function, and UM and MM represent single-peak and multi-peak, respectively. The waveform of the test function is shown in Fig 2.

**Table 1:** Test function information.

NO	Name	Range	Dim	f_min_	Type
F1	Sphere	[–100,100]	30	0	UM
F2	Schwefel2.22	[–10,10]	30	0	UM
F3	Schwefel 1.2	[–100,100]	30	0	UM
F4	Schwefel2.21	[–100,100]	30	0	UM
F5	Rosenbrock	[–30,30]	30	0	UM
F6	Step	[–100,100]	30	0	UM
F7	Quartic	[-1.28,1.28]	30	0	UM
F8	Schwefel	[–500,500]	30	-12569.487	MM
F9	Rastrigin	[-5.12,5.12]	30	0	MM
F10	Ackley	[–32,32]	30	0	MM
F11	Griewank	[–600,600]	30	0	MM
F12	Kowalik	[–5,5]	4	0.0003075	MM

Experimental simulation environment: All data in this paper are based on Intel processor with 2.60GHZ main frequency, 48G memory, and simulation software Matlab.

The swarm intelligence optimization algorithm solves the problem with certain randomness, in order to avoid the influence of randomness on the performance of the algorithm, all algorithms are run independently for 30 times. Tables 2–5 show the maximum value, optimal value, mean value and standard deviation of each algorithm after 30 runs, respectively. And Fridman test was performed for each algorithm, and the results are shown in Table 6.

**Table 2:** The results of 12 benchmarking functions of the maximum fitness function values.

Function	AOA	AO	HPO	SCSO	GWO	WOA	POA	DBO	MSIPOA
F1	1.2911e-25	1.3403e-103	3.1595e-170	9.6962e-114	1.9619e-27	5.7591e-73	8.6569e-98	1.771e-103	**0**
F2	**0**	4.2036e-49	8.9923e-89	1.9145e-58	3.01e-16	1.5211e-49	8.8113e-51	6.1486e-54	**0**
F3	0.078554	8.9882e-108	1.113e-147	2.8991e-94	0.00018464	82155.9206	1.2685e-97	6.6852e-38	**0**
F4	0.046059	1.5932e-52	1.8971e-74	7.9089e-50	1.3527e-06	85.9842	4.9618e-50	3.3021e-41	0
F5	28.9235	0.039579	26.0055	28.8599	28.738	28.8018	28.8233	26.781	28.7928
F6	3.7372	0.0067401	0.0013039	3.5163	1.2995	0.92639	3.97	0.2592	1.6457
F7	0.00036084	0.00063486	0.0010714	0.0010753	0.0052893	0.018885	0.00050673	0.0042543	0.0001093
F8	-4559.7389	-3673.4422	-7859.2195	-4615.7726	-3089.5025	-6606.9953	-6397.0175	-12565.4623	-12550.4416
F9	0	0	0	0	19.5329	0	0	22.8897	0
F10	8.8818e-16	8.8818e-16	8.8818e-16	8.8818e-16	1.4655e-13	7.9936e-15	4.4409e-15	8.8818e-16	8.8818e-16
F11	0.62208	0	0	0	0.026658	0.17399	0	0	0
F12	0.027023	0.00093881	0.020363	0.0012232	0.020363	0.0022368	0.020363	0.0014995	0.00044056

**Table 3:** The results of 12 benchmarking functions of the best (minimum) fitness function values.

Function	AOA	AO	HPO	SCSO	GWO	WOA	POA	DBO	MSIPOA
F1	2.9066e-142	1.829e-159	2.6788e-186	2.0996e-127	1.5994e-29	6.9441e-84	1.0997e-118	9.2728e-160	0
F2	0	1.0222e-80	3.0923e-98	3.1021e-66	2.1058e-17	1.6661e-58	1.8573e-59	1.2323e-83	0
F3	1.9875e-100	1.0771e-153	4.8166e-162	8.0139e-112	1.857e-09	30570.1814	1.6566e-122	1.1177e-146	0
F4	5.1955e-48	2.1137e-79	1.5564e-84	2.1129e-57	1.2876e-07	3.3174	1.9493e-59	3.3419e-75	0
F5	28.1097	0.00018233	22.8917	26.182	25.8956	27.0369	26.1726	25.3483	28.4775
F6	2.6083	2.5638e-06	5.7754e-10	1.0108	9.6028e-05	0.10023	1.5832	9.6592e-06	0.443
F7	2.5826e-06	4.7913e-06	1.5719e-05	7.5936e-06	0.00039774	0.00012947	2.4854e-05	0.00012452	1.3176e-06
F8	-6067.1507	-12566.9999	-10039.6296	-8117.7227	-7773.2131	-12568.952	-8787.9217	-12569.4865	-12569.4867
F9	0	0	0	0	5.6843e-14	0	0	0	0
F10	8.8818e-16	8.8818e-16	8.8818e-16	8.8818e-16	6.839e-14	8.8818e-16	8.8818e-16	8.8818e-16	8.8818e-16
F11	0.0095797	0	0	0	0	0	0	0	0
F12	0.00035726	0.00034184	0.00030749	0.00030749	0.0003075	0.00031137	0.00030749	0.00030749	0.00030167

**Table 4:** The results of 12 benchmarking functions in terms of the average fitness function values.

Function	AOA	AO	HPO	SCSO	GWO	WOA	POA	DBO	MSIPOA
F1	4.8143e-27	4.478e-105	1.0784e-171	6.0997e-115	5.2772e-28	2.6111e-74	2.8856e-99	5.9034e-105	0
F2	0	1.4548e-50	3.3623e-90	6.5948e-60	1.1167e-16	6.2238e-51	3.662e-52	2.0495e-55	0
F3	0.0080371	3.3532e-109	5.8034e-149	1.0745e-95	1.2227e-05	50242.0325	4.6125e-99	2.2284e-39	0
F4	0.026688	6.5025e-54	8.0563e-76	5.3453e-51	5.1603e-07	50.4373	2.6725e-51	1.1007e-42	0
F5	28.4631	0.0056265	23.722	28.1392	27.0899	28.0137	28.2578	25.7214	28.7042
F6	3.1787	0.00031334	4.3474e-05	1.9627	0.69205	0.45199	2.6828	0.015591	1.0373
F7	5.4735e-05	9.2772e-05	0.00022987	0.00014399	0.0019689	0.0036217	0.00019161	0.0014701	**3.9072e-05**
F8	-5282.9793	-7717.7658	-8976.238	-6471.1982	-6286.8108	-10371.6279	-7536.8766	-12567.8717	-12568.7571
F9	0	0	0	0	3.1577	0	0	1.7249	0
F10	8.8818e-16	8.8818e-16	8.8818e-16	8.8818e-16	1.0214e-13	4.4409e-15	4.0856e-15	8.8818e-16	8.8818e-16
F11	0.18276	0	0	0	0.0024988	0.015254	0	0	0
F12	0.0044615	0.00053639	0.0050952	0.00046127	0.0050797	0.00077741	0.0024968	0.00080143	0.00034449

**Table 5:** The results of the tested methods on 12 benchmarking functions of the STD values.

Function	AOA	AO	HPO	SCSO	GWO	WOA	POA	DBO	MSIPOA
F1	2.3642e-26	2.4468e-104	0	2.0559e-114	5.5053e-28	1.0809e-73	1.5805e-98	3.2334e-104	0
F2	0	7.6672e-50	1.6421e-89	3.4916e-59	7.2455e-17	2.7789e-50	1.6102e-51	1.1226e-54	0
F3	0.019807	1.6451e-108	2.297e-148	5.3056e-95	3.3458e-05	13774.3755	2.3174e-98	1.2206e-38	0
F4	0.020234	2.9591e-53	3.5397e-75	1.5019e-50	3.5667e-07	28.1682	9.7449e-51	6.0288e-42	0
F5	0.22398	0.0094771	0.62246	0.85109	0.73586	0.5132	0.70828	0.25822	0.089812
F6	0.29836	0.0012223	0.00023805	0.58726	0.3747	0.21444	0.498	0.058767	0.30506
F7	7.8423e-05	0.00011168	0.00021108	0.00022999	0.0012526	0.0041461	0.00012842	0.0011902	3.7124e-05
F8	384.9917	4012.196	521.3765	933.4594	954.3293	1840.5264	572.8175	2.57623	0.91655
F9	0	0	0	0	4.2817	0	0	5.4188	0
F10	0	0	0	0	2.1065e-14	2.6389e-15	1.084e-15	0	0
F11	0.17243	0	0	0	0.00681	0.047091	0	0	0
F12	0.0065147	0.00014583	0.0085729	0.00027192	0.0085791	0.00046203	0.0060663	0.00039387	3.3717e-05

**Table 6:** Results of Fridman test for all algorithms.

	AOA	AO	HPO	SCSO	GWO	WOA	POA	DBO	MSIPOA
Total average values	6.50	3.58	3.50	4.50	7.42	6.42	5.54	4.75	**2.79**
Total STD values	5.25	3.83	3.63	5.08	7.83	6.92	5.38	4.83	**2.25**
Final ranking	6	3	2	4	8	7	5	4	1

The worst values of the nine optimization algorithms are given in detail in Table 2, and it can be seen that MSIPOA has far better worst values than the other algorithms in 30 independent runs. In Tables 3–5, the specific values of optimal, mean and standard deviation are given in detail. From these tables, it can be seen that MSIPOA’s statistical results for the 12 tested functions are significantly better than the other algorithms.

MSIPOA searches for theoretical optimal values on F1, F2, F3, F4, F9 and F11. The AO algorithm achieves relatively better values on F5, and the HPO algorithm achieves better values on F6. Although MSIPOA does not achieve better values on these two test functions, it still has a significant improvement relative to the POA algorithm. On F8 and F12, although MSIPOA does not converge to the theoretical optimum, its converged solution is still the closest to the theoretical optimum among the seven intelligent algorithms. In F9 AOA, AO, HPO, SCSO, POA, and MSIPOA algorithms can achieve the theoretical optimal value. In F10 HPO, SCSO, DBO, and MSIPOA algorithms can achieve better value. In F11 AO, HPO, SCSO, WOA, POA, and MSIPOA algorithms can achieve the theoretical optimal value, in which HPO, SCSO, POA, and DBO algorithms are all new swarm intelligence algorithms proposed in 2022 with more robust performance, which also shows that these comparison algorithms are selected in this paper with sufficient comparison significance.

**Fig 3:**
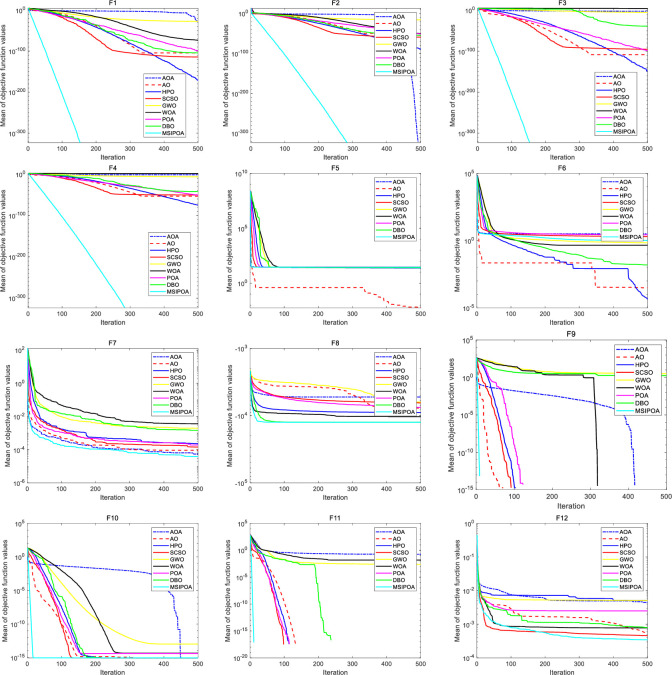
Convergence behavior of the algorithms based on 12 benchmark functions.

Fig 3 shows the average convergence curves of the seven algorithms under 12 benchmark test functions, and the convergence speed and convergence accuracy of each algorithm can be visualized through Fig 3. In F9, F10 and F11, although there are other algorithms that can converge to better results like MSIPOA, it can be visualized from Fig 3 that the convergence speed of MSIPOA is much better than other optimization algorithms. As can be seen from Fig 3 MSIPOA is still very competitive with them, demonstrating strong convergence speed and convergence accuracy on most of the tested functions.

**Fig 4:**
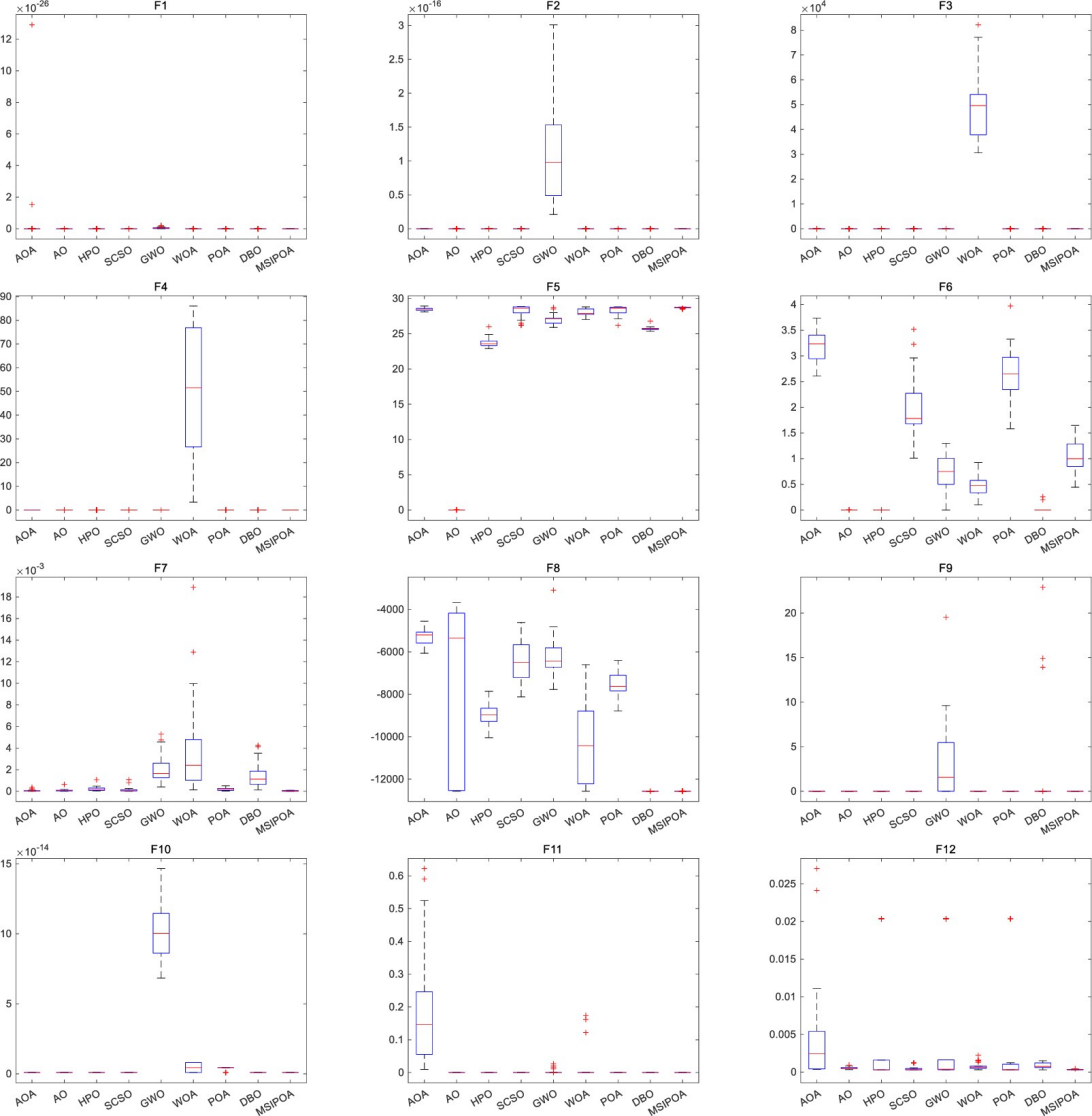
Box plot of the algorithms based on 12 benchmark functions.

Fig 4 shows the box plots of the iterative data for the results of the seven algorithms after 30 independent runs. The box plot’s top and bottom line segments indicate the data’s maximum and minimum values, respectively. The top and bottom edges of the box plot indicate the 75th percentile and 25th percentile, respectively. The thick line in the middle of the box plot indicates the median of the data. The box plot allows us to visualize the outliers of the data, the dispersion of the distribution, and the symmetry of the data. As can be seen in Fig 4, the MSIPOA algorithm has a very narrow box shape and maintains the lowest point in most of the tested functions. Compared with algorithms such as POA, the MSIPOA algorithm can get low box plots and no outliers. Compared with algorithms such as GWO, WOA, and SCSO, the MSIPOA algorithm has better optimization results. Although the HPO and DBO algorithms achieve relatively better results on F5 and F6, the MSIPOA algorithm for box plots achieves better results when the results of the 12 test functions are considered in an integrated manner.

The smaller the value obtained by Firdman test here, the better. By performing Firdman test on the total mean and standard deviation of the 12 test functions, the values obtained by MSIPOA are all optimal and the overall ranking is the first.

In summary, MSIPOA has apparent advantages over other swarm intelligence optimization algorithms in terms of convergence accuracy, convergence speed, and robustness, proving the MSIPOA algorithm’s excellent performance. In the next section, MSIPOA is used for image segmentation.

## 4. MSIPOA-based multi-threshold image segmentation

### 4.1. Experimental design

**Fig 5:**
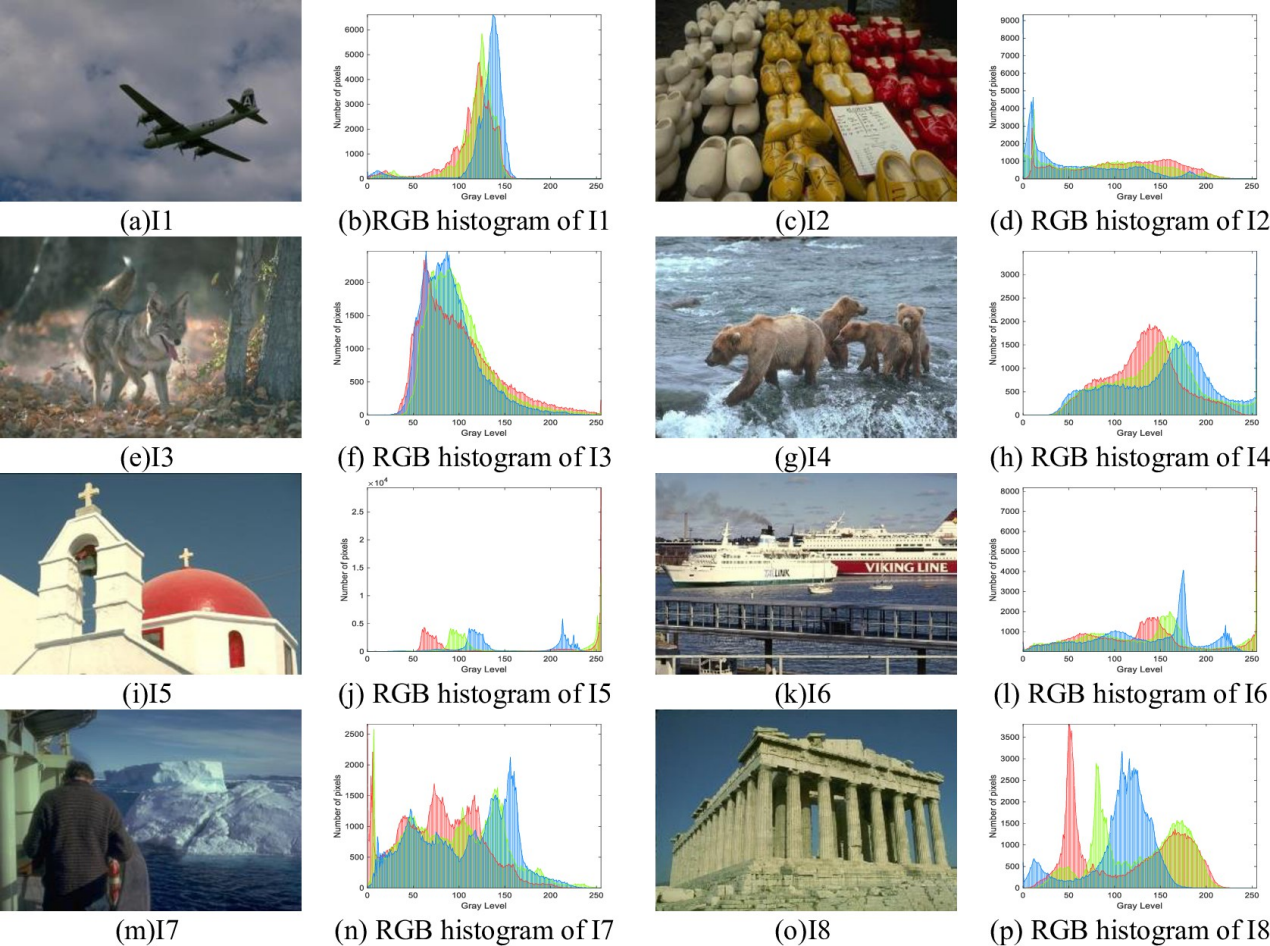
Image test set and RGB histogram.

The eight test images selected in this paper are from the Berkeley University BSDS300 image segmentation test set, the test image and its RGB histogram are shown in Fig 5. All algorithms have an initial population size of 30, a maximum number of iterations of 100, and 20 independent runs. The image segmentation thresholds are set to 2, 4, and 6, respectively.

### 4.2. Image segmentation quality metrics

In this paper, the metrics to measure the effectiveness of image segmentation are the feature similarity index (FSIM), structural similarity index (SSIM), and peak signal-to-noise ratio (PSNR).

PSNR, mainly with the help of the error of the corresponding pixel points of the image, has been most commonly used in recent years in the objective evaluation index of image quality, which is mathematically defined as:

$$PSNR=20\times \log_{10}\frac{2^{n}}{MSE}$$ (13)

In the above equation, n is usually 8, MSE indicates the expected value of the squared difference between the estimated and actual values, and PSNR is measured in dB. The larger the PSNR value, the less noise rest of the image, indicating good noise resistance of the segmented image and good image segmentation effect.

SSIM is a metric used to measure the similarity of two images before and after compression. SSIM divides the image information into three contrast modules, namely luminance (l), contrast (c), and structure (s). Assuming that the images before and after segmentation are x and y, respectively, SSIM is defined as:

$$SSIM(x,y)={\left[l(x,y)\right]}^{\alpha }\cdot {\left[c(x,y)\right]}^{\beta }\cdot {\left[s(x,y)\right]}^{\gamma }$$ (14)

*α*, *β*, *γ* are the coefficients of the three comparison modules greater than 0, respectively, and the three comparison modules are defined specifically as:

$$l(x,y)=\frac{2\mu_{x}\mu_{y}+C_{1}}{\mu_{x}^{2}+\mu_{y}^{2}+C_{1}}$$ (15)

$$c(x,y)=\frac{2\delta_{x}\delta_{y}+C_{2}}{\delta_{x}^{2}+\delta_{y}^{2}+C_{2}}$$ (16)

$$s(x,y)=\frac{\delta_{xy}+C_{3}}{\delta_{x}\delta_{y}+C_{3}}$$ (17)

Where *C*_1_,*C*_2_,*C*_3_ is a constant and *μ*_*x*_, *μ*_*y*_ is all pixels of the image, *δ*_*x*_, *δ*_*y*_ represents the standard deviation of the image pixel values, and *δ*_*xy*_ represents the covariance of the two images. In practical applications *α* = *β* = *γ* = 1, *C*_3_ = 0.5*C*_2_. Then the expression of SSIM is:

$$SSIM(x,y)=\frac{2\mu_{x}\mu_{y}+C_{1}}{\mu_{x}^{2}+\mu_{y}^{2}+C_{1}}\cdot \frac{2\delta_{xy}+C_{2}}{\delta_{x}^{2}+\delta_{y}^{2}+C_{2}}$$ (18)

The value of SSIM ranges from 0 to 1. The larger the value, the smaller the difference between the two images, the smaller the image segmentation quality, and the better the image segmentation effect.

FSIM considers that not all pixels in an image have the same importance. For example, the pixel points at the edge of a part of an object in an image are more important for defining its structure. FSIM uses two features, phase consistency feature (PC) and gradient feature (GM), where PC can portray the local structure of an image and GM can extract the changes in an image. A more significant FSIM value indicates that the test image is closer to the reference image.

The similarity of PC is calculated as follows:

$$S_{PC}(x)=\frac{2PC_{1}(x)*PC_{2}+T_{1}}{PC_{1}^{2}(x)+PC_{2}^{2}(x)+T_{1}}$$ (19)

The similarity of GM is calculated as follows:

$$S_{G}(x)=\frac{2G_{1}(x)*G_{2}(x)+T_{2}}{G_{1}^{2}(x)+G_{2}^{2}(x)+T_{2}}$$ (20)

The formula for FSIM is as follows:

$$FSIM=\frac{\sum {}_{x\in \Omega }S_{L}(x)*PC_{m}(x)}{\sum {}_{x\in \Omega }PC_{m}(x)}$$ (21)

where *α*, *β* is generally taken as 1, $PC_{m}(x)=\max(PC_{1}(x),PC_{2}(x))$.

### 4.3. Simulation and results

**Fig 6:**
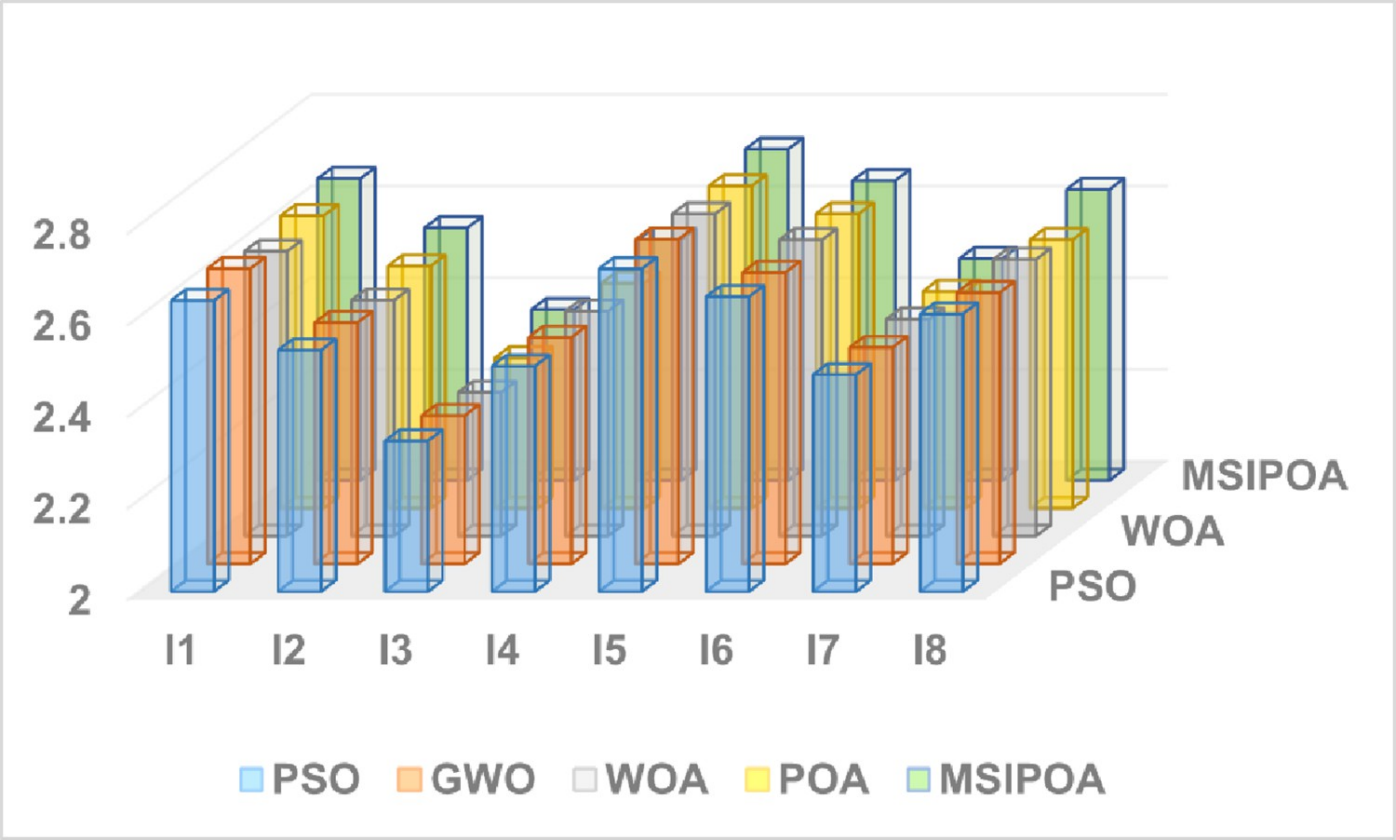
Summary of the FSIM results for the all algorithms at all levels.

**Fig 7:**
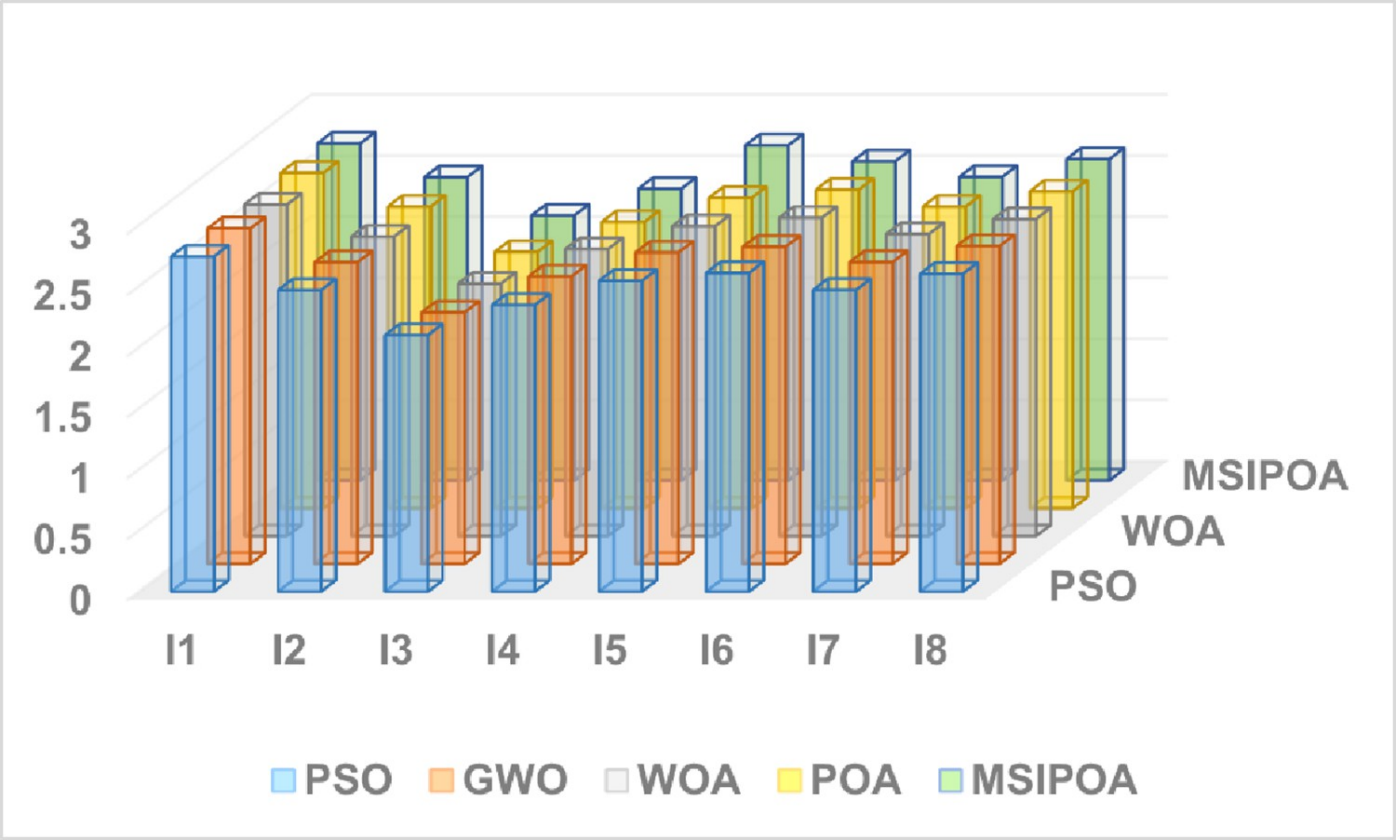
Summary of the SSIM results for the all algorithms at all levels.

**Fig 8:**
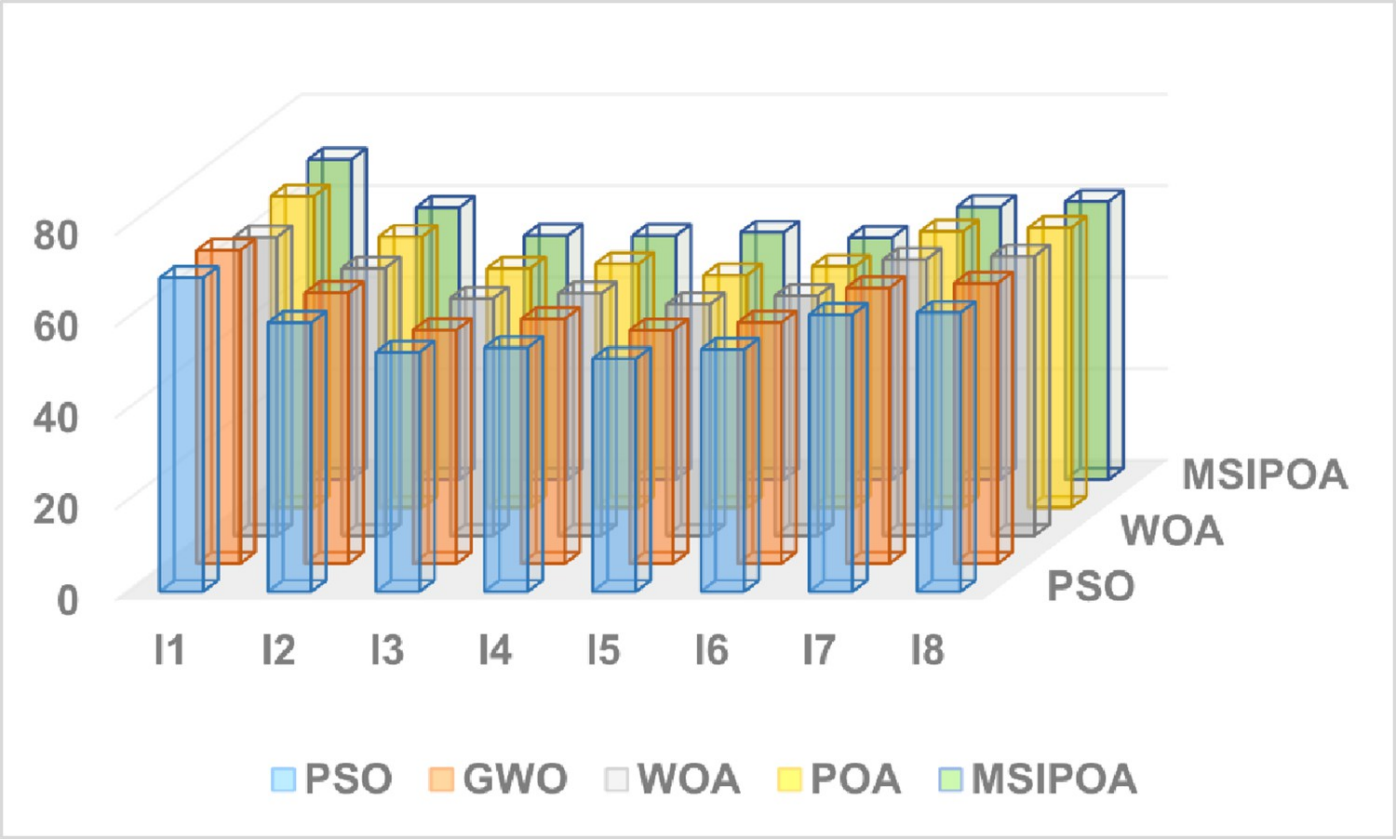
Summary of the PSNR results for the all algorithms at all levels.

**Fig 9:**
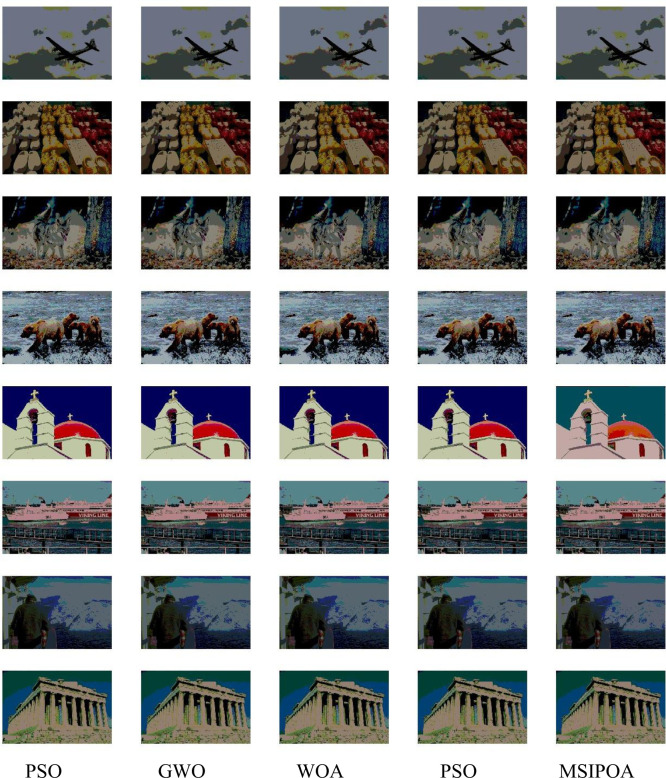
2-threshold image segmentation results.

**Fig 10:**
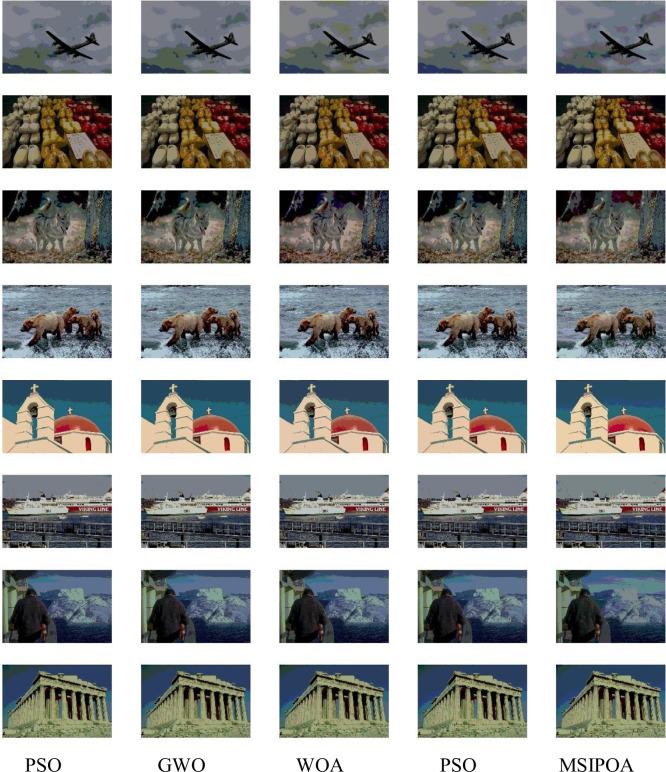
4-threshold image segmentation results.

**Fig 11:**
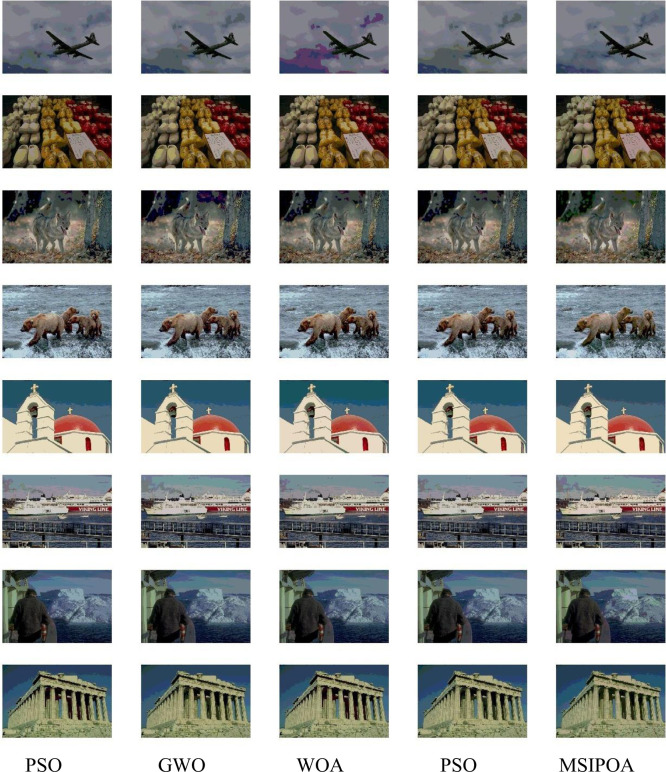
6-threshold image segmentation results.

This paper selects symmetric cross-entropy multi-threshold image segmentation algorithms based on PSO [35], WOA, GWO, POA, and MSIPOA for comparison. Figs 6–8 show the sum of the fitnesses under all thresholds for the different test sets corresponding to each algorithm, respectively. Figs 9–11 show the image segmentation results for different algorithms with 2, 4 and 6 thresholds, respectively. Tables 7–9 show the numerical evaluation results of FSIM, SSIM, and PSNR for image segmentation, respectively.

**Table 7:** Results of the FSIM measure for all algorithms.

		PSO	GWO	WOA	POA	MSIPOA
2	I1	0.86667	0.86717	0.86094	0.86689	**0.86904**
	I2	0.75482	0.75541	0.75566	0.75491	**0.75715**
	I3	0.68263	0.68246	0.68343	0.68208	**0.68764**
	I4	0.73506	0.73534	0.73556	0.73553	**0.74106**
	I5	0.86528	0.86525	0.865	0.86486	**0.87862**
	I6	0.81036	0.81073	0.81049	0.81089	**0.81368**
	I7	0.73326	0.73254	0.73283	0.73242	**0.73643**
	I8	0.78126	0.78176	0.78324	0.78172	**0.7872**
4	I1	0.87273	0.87821	0.87451	0.87384	**0.88187**
	I2	0.8684	0.86211	0.85817	0.8622	**0.873**
	I3	0.7921	0.79326	0.77533	0.79269	**0.81311**
	I4	0.85092	0.85194	0.85166	0.85078	**0.86795**
	I5	0.91172	0.91237	0.90996	0.9113	**0.91332**
	I6	0.90087	0.90002	0.90129	0.90061	**0.90451**
	I7	0.84742	0.84705	0.84745	0.84674	**0.84893**
	I8	0.89439	0.89488	0.89256	0.89107	**0.91474**
6	I1	0.89578	0.89931	0.88591	0.89806	**0.90802**
	I2	0.89904	0.90922	0.90078	0.91153	**0.92139**
	I3	0.85429	0.84749	0.85489	0.85339	**0.87253**
	I4	0.90614	0.90716	0.9035	0.90536	**0.91721**
	I5	0.92755	0.9307	0.92822	0.92719	**0.93154**
	I6	0.9323	0.92548	0.93478	0.93106	**0.93659**
	I7	0.89263	0.89244	0.89313	0.8943	**0.89907**
	I8	0.93056	0.91521	0.92799	0.91342	**0.9331**

**Table 8:** Results of the SSIM measure for all algorithms.

		PSO	GWO	WOA	POA	MSIPOA
2	I1	0.88182	0.88205	0.87237	**0.88293**	0.88115
	I2	0.71652	0.71736	0.71714	0.71745	**0.71867**
	I3	0.55649	0.55514	0.55709	0.55605	**0.56266**
	I4	0.65193	0.65229	0.65176	0.65183	**0.65956**
	I5	0.67754	0.67755	0.67764	0.67685	**0.86558**
	I6	0.79062	0.79026	0.79062	0.79175	**0.79285**
	I7	0.72639	0.72209	0.72338	0.72209	**0.72729**
	I8	0.78438	0.78438	0.78267	0.7824	**0.78882**
4	I1	0.91779	0.91894	0.92042	0.91952	**0.92517**
	I2	0.85444	0.85121	0.84362	0.85078	**0.85763**
	I3	0.71847	0.71678	0.68432	0.71824	**0.75921**
	I4	0.80748	0.80962	0.81178	0.80637	**0.8335**
	I5	0.92393	0.92541	0.92169	0.92384	**0.93007**
	I6	0.88476	0.88617	0.88512	0.88447	**0.89075**
	I7	0.84185	0.84199	0.84229	0.84117	**0.85373**
	I8	0.89461	0.89474	0.89347	0.8925	**0.90946**
6	I1	0.94345	0.94509	0.92092	0.94586	**0.95325**
	I2	0.8966	0.90033	0.8905	0.90161	**0.9079**
	I3	0.82433	0.78645	0.82547	0.82443	**0.84824**
	I4	0.88683	0.89157	0.88551	0.88667	**0.89525**
	I5	0.94226	0.946	0.93766	0.94009	**0.949**
	I6	**0.93277**	0.91858	0.93024	0.93124	0.93021
	I7	0.90094	0.90551	**0.90725**	0.90634	0.90576
	I8	0.92206	0.9223	0.9187	0.91916	**0.93328**

**Table 9:** Results of the PSNR measure for all algorithms.

		PSO	GWO	WOA	POA	MSIPOA
2	I1	18.2422	18.2282	18.1746	18.2108	**18.4223**
	I2	16.3286	16.3536	16.3598	16.3225	**16.3796**
	I3	14.2272	14.2362	14.2341	14.241	**14.2454**
	I4	13.9505	13.9539	13.9248	13.9256	**13.9833**
	I5	12.0759	12.0911	12.0951	12.0678	**13.4647**
	I6	13.9114	13.8859	13.8988	13.8987	**13.9652**
	I7	**16.4984**	16.3858	16.4321	16.4126	16.3074
	I8	**16.153**	16.1188	16.1025	16.1003	16.0962
4	I1	23.7182	23.6232	22.973	23.7515	**23.953**
	I2	20.1323	20.104	19.8729	20.1579	**20.2924**
	I3	17.5055	17.2976	16.9709	17.4901	**18.4634**
	I4	18.2797	18.3389	18.2407	18.3324	**18.3745**
	I5	17.6596	17.699	18.1002	17.711	**18.4149**
	I6	18.1034	**18.1691**	17.9748	18.0631	18.0463
	I7	20.6546	20.7237	20.6435	20.6485	**20.8553**
	I8	21.2015	21.1213	21.2648	21.1869	**21.321**
6	I1	26.719	26.7033	24.1925	26.1916	**27.6478**
	I2	22.4023	22.795	22.3081	22.8391	**22.8618**
	I3	20.6719	19.6017	**20.7344**	20.65	20.6955
	I4	21.1675	**21.2691**	20.847	21.0799	21.0221
	I5	21.2164	21.2624	20.434	21.0935	**22.2278**
	I6	20.961	20.7204	20.6265	20.8094	**20.9791**
	I7	**23.4652**	23.1979	23.3349	23.3819	22.5565
	I8	23.7134	**24.1183**	23.7891	23.9617	23.4314

Tables 7–9 shows the average FSIM, SSIM, and PSNR of the segmented images after testing the algorithm. The table shows that the FSIM, SSIM, and PSNR values of the segmented images of each algorithm show an increasing trend as the threshold value gradually increases, and the peak signal-to-noise ratio, structural similarity, and feature similarity before and after image segmentation are gradually becoming higher. As can be seen from Tables 3–5, the MSIPOA achieved optimal mean values for FSIM, SSIM and PSNR metrics of 100%, 87.5% and 70.83%, respectively. This fully illustrates the advantages that the FSIM, SSIM, and PSNR of images segmented based on MSIPOA symmetric cross-entropy show compared with other algorithms, especially in its lowest distortion degree of images before and after segmentation, which greatly ensures the similarity between the two images before and after segmentation. The differences between the FSIM, SSIM, and PSNR values of MSIPOA and other algorithms become increasingly apparent as the threshold value increases. It shows that the segmented image obtained after solving the optimal threshold using MSIPOA is closest to the original image, which retains more information about the original image and reflects the excellent global search ability of the MSIPOA algorithm.

The results of the three metrics after threshold partitioning by each algorithm are subjected to Fridman test, where the larger the value is, the better it is. Table 10 shows the specific values after Fridman test, and the MSIPOA metrics are optimal.

**Table 10:** Results of Fridman test for all algorithms.

	PSO	GWO	WOA	POA	MSIPOA
FSIM	2.4167	2.7917	2.6667	2.1250	**5.0000**
SSIM	2.6667	2.8333	2.3542	2.4792	**4.6667**
PSNR	3.1667	2.9167	2.1667	2.7083	**4.0417**

The experimental results show that the MSIPOA algorithm has the characteristics of solid self-adaptation, fast finding speed, and high finding accuracy and can be applied to the image segmentation problem. The MSIPOA algorithm proposed in this paper outperforms the segmentation performed directly using PSO, GWO, WOA, and POA algorithms regarding quality. Its solution to the multi-threshold image segmentation problem is more advantageous to obtain a more accurate segmented image. The results on the Berkeley dataset also show the effectiveness and robustness of the proposed algorithm.

## 5. Conclusion

In response to the problems of low accuracy, slow convergence, and high computational complexity of traditional multi-threshold segmentation methods, this study proposes a color image segmentation algorithm based on the MSIPOA algorithm. To address the limitations of POA, Sine chaos mapping, levy flight strategy, spiral search strategy, and the strategy of fusion sine cosine optimization algorithm are introduced to improve it. Then, the symmetric cross-entropy sum of images is used as the fitness function to search the optimal segmentation threshold of images quickly and precisely using the hunting behavior of pelican populations. To verify the effectiveness of the proposed MSIPOA, the convergence performance is tested on 12 benchmark test functions with the new algorithms, such as DBO, SCSO, and POA. The experimental results show that the MSIPOA algorithm outperforms other optimization algorithms in terms of convergence speed and convergence accuracy. The experimental comparison with classical algorithms such as PSO, WOA, and GWO for image segmentation shows that the FSIM, SSIM, and PSNR metrics of MSIPOA achieve optimal average values of 100%, 87.5%, and 70.83%, respectively, with significantly better results than the symmetric cross-entropy results of PSO, GWO and POA algorithms. The segmented images obtained based on the MSIPOA algorithm are of higher quality, and the algorithm runs more stably.

### 5.1. Limitations and future works

In this paper, we propose a color image segmentation algorithm based on the MSIPOA algorithm, and the effect of this algorithm image segmentation is better than other methods through simulation testing. However, there are still some limitations in this paper, i.e., it is not combined with practical applications.

In the next step, we intend to integrate it with practical applications. For example, CT analysis in medicine, IoT task scheduling, industrial defect detection and data pre-processing
